# Advances in Hip Replacement Surgery

**DOI:** 10.3390/jcm12103439

**Published:** 2023-05-12

**Authors:** William G. Blakeney, Markus Kuster

**Affiliations:** 1 Department of Orthopaedic Surgery, Royal Perth Hospital, Wellington St., Perth, WA 6000, Australia; markus.kuster@health.wa.gov.au; 2 Department of Surgery, University of Western Australia, 35 Stirling Hwy, Crawley, WA 6009, Australia; 3 Department of Orthopaedic Surgery, Sir Charles Gairdner Hospital, Perth, WA 6000, Australia

Total hip arthroplasty (THA) is one of the most successful types of surgical operation, with some considering it “the operation of the century” [1]. Early attempts at THA were beset with problems due to inadequate fixation in the bone and significant soft tissue reactions to poorly performing bearing materials, with resultant early failure. Sir John Charnley, through the development of his low-friction arthroplasty method in 1971, transformed the outcomes for THA with survivorship rising to approximately 80% at 25 years [2]. Since then, continuing advances in the design, materials, and surgical techniques used in THA have led to much-improved survivorship and clinical outcomes.

Patients’ expectations regarding life following THA have therefore changed. They are concerned not just about survivorship, but also about quality of life issues, often wishing to continue with their high-activity vocational and recreational interests. Today, it is not uncommon for post-operative patients to experience a natural-feeling joint or “forgotten joint” [3].

Many recent advances in hip arthroplasty aim to reduce the small number of patients who are unsatisfied with the outcomes of their surgery or experience complications. This Special Issue of the *Journal of Clinical Medicine* looks at recent advances in hip replacement surgery.

The concept of personalised joint replacement tailored to the patient, rather than a standardised “one-size-fits-all” approach, has recently come to the fore. Personalised THA should aim to restore functional biomechanics, with the stress transfer from implant to bone mimicking physiological forces. It should feel like a “forgotten joint” with maximised impingement-free range of motion and stability [4]. Bearing wear should be minimal to provide lifetime implant survivorship and enable unrestricted activities. Finally, patients should have a rapid and complication-free recovery from the surgery.

In their article “Personalized Hip Joint Replacement with Large Diameter Head: Current Concepts”, Venditolli et al. explore the use of either monobloc or dual-mobility large-diameter-head (LDH) THA to achieve a greater range of motion with a reduced risk of dislocation [5]. The larger head–neck offset allows for a supraphysiologic range of motion (ROM), which can compensate for the patient’s abnormal spinopelvic mobility and surgical imprecision. The authors describe how appropriate biomechanical reconstruction can restore normal gait parameters. This results in unrestricted activities and higher patient satisfaction scores. The authors recommend large-diameter ceramic-on-ceramic for younger patients and dual-mobility bearings for others. The use of dual-mobility implants for all patients has also previously been suggested [6]. A registry analysis by Di Martino et al. looked at revisions for instability, and demonstrated that dual-mobility implants showed a significantly lower risk of re-revision for dislocation compared to standard cups [7].

Pallazzuolo et al. looked at another LDH option in their meta-analysis, arguing that resurfacing hip arthroplasty is a safe and effective alternative to THA in young patients [8]. Metal-on-metal THA has largely been abandoned due to complications caused by metal ions. Wakabayashi et al. report their 10-year results of primary THA using a modular metal-on-metal acetabular prosthesis, which led to a 29% prevalence of pseudotumours [9].

Recognition of the importance of spinopelvic alignment and how this affects a surgeon’s positioning of the components is another area in which considerable advances have been made. Sivaloganathan et al. discuss the evolution of concepts in functional acetabular orientation in their review article, “Can Personalized Hip Arthroplasty Improve Joint Stability?” [10]. In particular, the authors focus on understanding and using dynamic pre-operative planning to achieve the kinematic alignment technique for THA.

This personalisation of implant positioning necessitates increased exactness of pre-operative planning. The execution of surgery therefore requires precision tools, which have been developed in recent years. Technology that improves the precision of implantation—robotics, navigation, and patient-specific instrumentation (PSI)—has allowed for the development of personalised alignment strategies, as well as a reduction in surgical outliers. Robotic THA has been the focus of many contemporary advances in hip surgery. Bullock et al., in their review article “Robotics in Total Hip Arthroplasty: Current Concepts”, explore this new technology [11]. They look at the history of robotics in THA, the current systems in operation, as well as their potential in the future. They synthesise the current clinical evidence, which shows that robotic THA improves radiological outcomes; however, a longer-term follow-up stating that this confers improved functional outcomes has yet to be conducted.

Another advancement in THA is explored in the article “Modernizing Total Hip Arthroplasty Perioperative Pathways: The Implementation of ERAS-Outpatient Protocol” [12]. The Enhanced Recovery After Surgery (ERAS) principles aim to reduce the duration of a patient’s hospital stay by delivering optimized patient-centred care through a multi-disciplinary team, with the ultimate aim of achieving a “pain and risk-free operation”. The authors delineate the core aspects of the ERAS protocol, which incorporate preoperative patient education and medical optimization, perioperative improved pain control, blood conservation, nutritional support, early mobilization, the maintenance of gastrointestinal function, and a reduction in adverse events.

We hope that the benefits that arise from advances in hip replacement surgery will lead to THA also being considered the operation of the next century.
